# Early fatigue in cancer patients receiving PD-1/PD-L1 checkpoint inhibitors: an insight from clinical practice

**DOI:** 10.1186/s12967-019-02132-x

**Published:** 2019-11-15

**Authors:** Alessio Cortellini, Maria G. Vitale, Federica De Galitiis, Francesca R. Di Pietro, Rossana Berardi, Mariangela Torniai, Michele De Tursi, Antonino Grassadonia, Pietro Di Marino, Daniele Santini, Tea Zeppola, Cecilia Anesi, Alain Gelibter, Mario Alberto Occhipinti, Andrea Botticelli, Paolo Marchetti, Francesca Rastelli, Federica Pergolesi, Marianna Tudini, Rosa Rita Silva, Domenico Mallardo, Vito Vanella, Corrado Ficorella, Giampiero Porzio, Paolo A. Ascierto

**Affiliations:** 1 Medical Oncology Unit, St. Salvatore Hospital, L’Aquila, Italy; 2 Department of Biotechnology and Applied Clinical Sciences, University of L’Aquila, Via Vetoio, 67100 L’Aquila, Italy; 3 Medical Oncology, University Hospital of Modena, Modena, Italy; 4 Istituto Dermopatico dell’immacolata, IDI-IRCCS, Rome, Italy; 5 Department of Clinical and Molecular Medicine, “Sapienza” University of Rome, Rome, Italy; 6 Oncology Clinic, Università Politecnica delle Marche, Ospedali Riuniti di Ancona, Ancona, Italy; 7 Department of Medical, Oral & Biotechnological Sciences University G. D’Annunzio, Chieti-Pescara, Chieti, Italy; 8 Clinical Oncology Unit, S.S. Annunziata Hospital, Chieti, Italy; 9 Medical Oncology, Campus Bio-Medico University, Rome, Italy; 10 Medical Oncology (B), Policlinico Umberto I, “Sapienza” University of Rome, Rome, Italy; 11 Medical Oncology, Fermo Area Vasta 4, Fermo, Italy; 12 Medical Oncology, AV2 Fabriano ASUR Marche, Fabriano, Italy; 13 Melanoma, Cancer Immunotherapy and Development Therapeutics Unit, Istituto Nazionale Tumori-IRCCS Fondazione “G. Pascale”, Naples, Italy

**Keywords:** Fatigue, Cancer, Immune-related adverse events, IL-6, PD-1/PD-L1 inhibitors, Immunotherapy

## Abstract

**Background:**

Fatigue was reported as the most common any-grade adverse event (18.3%), and the most common grade 3 or higher immune-related adverse event (irAE) (0.89%) in patients receiving PD-1/PD-L1 checkpoint inhibitors in clinical trial.

**Methods:**

The aim of this retrospective multicenter study was to evaluate the correlations between “early ir-fatigue”, “delayed ir-fatigue”, and clinical outcomes in cancer patients receiving PD-1/PD-L1 inhibitors in clinical practice.

**Results:**

517 patients were evaluated. After the 12-weeks landmark selection, 386 (74.7%) patients were eligible for the clinical outcomes analysis. 40.4% were NSCLC, 42.2% were melanoma, 15.3% renal cell carcinoma and 2.1% other malignancies. 76 patients (19.7%) experienced early ir-fatigue (within 1 month from treatment commencement), while 150 patients (38.9%) experienced delayed ir-fatigue. Early ir-fatigue was significantly related to shortened PFS (HR = 2.29 [95% CI 1.62–3.22], p < 0.0001) and OS (HR = 2.32 [95% CI 1.59–3.38], p < 0.0001) at the multivariate analysis. On the other hand, we found a significant association between the occurrence of early ir-fatigue, ECOG-PS ≥ 2 (p < 0.0001), and disease burden (p = 0.0003). Delayed ir-fatigue was not significantly related to PFS nor OS.

**Conclusions:**

Early ir-fatigue seems to be negative prognostic parameter, but to proper weight its role we must to consider the predominant role of performance status, which was related to early ir-fatigue in the study population.

## Introduction

Immune checkpoint inhibitors (ICIs) are characterized by a distinctive side effect profile, compared to other anticancer drugs. The adverse events occurring during ICIs are collectively named as immune-related adverse events (irAEs). IrAEs mimic autoimmune diseases by definition, leading to a dysfunction of peripheral T-cells tolerance, where immune checkpoints play a pivotal role [1, 2]. However, in clinical practice the immunological basis of each adverse event occurring during ICIs is an assumption, as if the underlying mechanism is always putatively immune-related.

A recent systematic review and meta-analysis has summarized the incidence and grade of irAEs across clinical trials with anti-PD-1/PD-L1 (programmed death-1/programmed death-ligand 1) agents [3]. Fatigue was reported as the most common any-grade adverse event (18.3%), and the most common grade 3 or higher irAE (0.89%) [3]. Considering the recent evidences suggesting that the occurrence of common irAEs (such as cutaneous irAEs, endocrine irAEs and gastro-intestinal irAEs) might be considered a surrogate predictor of clinical benefit with ICI [4, 5], it would be interesting to investigate the clinical implications of the occurrence of immune-related fatigue (ir-fatigue) in clinical practice. We performed the present analysis in order to explore and weighing the role of ir-fatigue occurrence in cancer patients receiving PD-1/PD-L1 checkpoint inhibitors.

## Materials and methods

This analysis was performed within the already available “real-life” multicenter retrospective data set, where we collected clinical data of advanced cancer patients who underwent treatment with single PD-1/PD-L1 checkpoint inhibitors as first or subsequent line [5–7] (University of L’Aquila, Internal Review Board protocol number 32865, approved on July 24th, 2018). Only patients with data availability regarding ir-fatigue were included in the present analysis.

The aim of this analysis was to evaluate the correlations between “early ir-fatigue”, “delayed ir-fatigue”, and following clinical outcomes: objective response rate (ORR), progression free survival (PFS) and overall survival (OS). In order to minimize the negative selection effect that the experienceness of fatigue may have regarding poorer clinical condition, a landmark of 12 weeks was used to perform all the efficacy analysis; all the patients whit a follow-up for PFS shorter than 12 weeks were excluded (regardless of progression events). We chose 12 weeks because of being the preferred landmark for patients enrollment in prospective clinical trials [8]. Early ir-fatigue was defined as the occurrence of any grade fatigue within the first month form the immunotherapy commencement, while delayed ir-fatigue was defined as the occurrence of any grade fatigue after the first month from the immunotherapy commencement. Ir-fatigue and irAEs overall were graded according to the National Cancer Institute Common Terminology Criteria for Adverse Events (CTCAE; version 4.0) and cumulatively reported as crude incidence.

Median period of follow-up was calculated according to the reverse Kaplan–Meier method.

Median PFS and median OS were evaluated using the Kaplan–Meier method. Chi square was used to correlate ORR and ir-fatigue (early and delayed). Cox regression was used for the univariate analysis of PFS and OS according to early and delayed ir-fatigue. A multivariate Cox regression was used to evaluate those parameters which resulted to be significant at the univariate analysis. In order to properly weighing the impact on clinical outcomes and to find appropriate covariates, the correlations between ir-fatigue, and baseline clinical factors (age, ECOG-PS [Easter Cooperative Oncology Group-Performance Status], sex, burden of disease and treatment line) were evaluated with the Chi square test. Baseline factors which were significantly related to ir-fatigue were not included in the multivariate analyses [9]. The fatigue reporting across different tumor types can widely vary [10], then, given to its role, primary tumor was included in multivariate analysis regardless of its significance at univariate analysis. Moreover, a correlation analysis of ECOG-PS and ir-fatigue, and an efficacy analyses according to the experience of early and delayed ir-fatigue, were separately performed in melanoma and non small cell lung cancer (NSCLC) cohorts. All statistical analyses were performed using MedCalc Statistical Software version 19.0.4 (MedCalc Software bvba, Ostend, Belgium; https://www.medcalc.org; 2019).

## Results

517 patients had data availability regarding ir-fatigue; treatment commencement ranged from June 2014 to April 2019. After the 12-weeks landmark selection, 386 (74.7%) patients were eligible for the clinical outcomes analysis. Table 1 summarized all the patients features 40.4% were NSCLC, 42.2% were melanoma, 15.3% renal cell carcinoma and 2.1% other malignancies. 67.4% of the included patients were male. The median age was 68 years and 170 patients (44.0%) were elderly (≥ 70 yo). 48 patients (12.4%) had an ECOG-PS ≥ 2, 167 patients (43.3%) had more than 2 metastatic sites and 137 patients (35.5%) received PD-1/PD-L1 inhibitors as first line. 213 patients (55.2%) experienced any grade irAEs, while 46 patients (11.9%) experienced G3/G4 irAEs. 76 patients (19.7%) experienced early ir-fatigue: 2 of them (2.6%) G3/G4 early ir-fatigue, while 74 (97.4%) G1/G2 early ir-fatigue. 150 patients (38.9%) experienced delayed ir-fatigue: 7 of them (4.7%) G3/G4 early ir-fatigue, while 143 (95.3%) G1/G2 delayed ir-fatigue. 61 patient (15.8%) experienced both early and delayed ir-fatigue.

**Table 1:** Patients characteristics

	N° (%)
	386
Age (years)	
Median	68
Range	21–88
Elderly (≥ 70)	170 (44)
Sex	
Male	260 (67.4)
Female	126 (32.6)
ECOG PS	
0–1	338 (87.6)
≥ 2	48 (12.4)
Primary tumor	
NSCLC	156 (40.4)
Melanoma	163 (42.2)
Renal cell carcinoma	59 (15.3)
Others	8 (2.1)
No. of metastatic sites	
≤ 2	219 (56.7)
> 2	167 (43.3)
Type of anti-PD-1/PD-L1 agent	
Pembrolizumab	120 (31.1)
Nivolumab	253 (65.5)
Atezolizumab	10 (2.6)
Others	3 (0.8)
Treatment line of immunotherapy	
First	137 (35.5)
Non-first	249 (64.5)

Among the 382 evaluable patients 173 response of disease were observed and the ORR was 45.3% (95% CI 38.7–52.5). The ORR among patients who experienced early ir-fatigue was 36% (95% CI 23.7–52.3), while among patients who did not experienced early ir-fatigue was 47.5% (95% CI 40.1–55.9), without statistically significant difference (p = 0.0718). The ORR among patients who experienced delayed ir-fatigue was 42.9% (95% CI 33.1–54.8), while among patients who did not experienced delayed ir-fatigue was 46.8% (95% CI 38.4–56.4), without statistically significant difference (p = 0.4641).

**Fig. 1:**
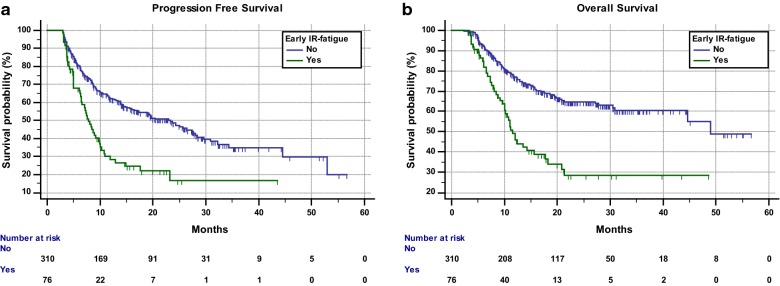
Kaplan–Meier survival curves according to early ir-fatigue. **a** Progression free survival. **b** Overall survival

The median follow up was 21.2 months. Median PFS and median OS in the overall population were 16.5 months (95% CI 12.3–23.1; 201 events) and 44.6 months (95% CI 30.4–48.9; 248 censored patients), respectively. Median PFS of patients who experienced early ir-fatigue was 7.8 months (95% CI 6.4–9.7; 52 events), while median PFS of patients who did not was 23.4 months (95% CI 15.9–27.7; 149 events) whit a statistically significant difference (HR = 2.25 [95% CI 1.63–3.1], p < 0.0001) (Fig. 1a). Patients who experienced early ir-fatigue had a median OS of 11.5 months (95% CI 10–17.7; 32 censored patients), while patients who did not experienced early ir-fatigue had a median OS of 48.9 months (95% CI 44.6–48.9; 216 censored patients) with a statistically significant difference (HR = 2.63 [95% CI 1.83–3.77], p < 0.0001) (Fig. 1b). Patients who experienced delayed ir-fatigue had a median PFS of 14.4 months (95% CI 10.5–23.4; 85 events), while patients who did not experienced delayed ir-fatigue had a median PFS of 17.1 months (95% CI 11.8–24.3; 116 events) (HR = 1.11 [95% CI 0.83–1.46], p = 0.4752). Median OS of patients who experienced delayed ir-fatigue was 30.5 months (95% CI 18.7–44.6; 87 censored patients), while median OS of patients who did not was 48.9 months (95% CI 48.9–48.9; 161 censored patients) without a statistically significant difference (HR = 1.28 [95% CI 0.91–1.79], p = 0.1431).

Among patients who experienced early ir-fatigue 21 (27.6%) had an ECOG-PS ≥ 2, while 27 (8.7%) patients had an ECOG-PS ≥ 2 among patients who did not experienced early-ir-fatigue (p < 0.0001). Similarly, 47 patients (61.8%) among those who experienced early ir-fatigue, had more than 2 metastatic sites, while 120 patients (38.7%) had more than 2 metastatic sites among patients who did not experienced ir-fatigue (p = 0.0003).

We did not find significant associations between early ir-fatigue and patients sex, age (elderly vs non-elderly), and treatment line (first vs non-first) (data not shown). Table 2 summarized univariate and multivariate analyses of PFS, while Table 3 summarized univariate and multivariate analyses of OS; early ir-fatigue was confirmed an independent predictor for shorter PFS (HR = 2.29 [95% CI 1.62–3.22], p < 0.0001), and shorter OS (HR = 2.32 [95% CI 1.59–3.38], p < 0.0001).

**Table 2:** Univariate and multivariate analyses of PFS

Variable (comparator)	Progression free survival			
	Univariate analysis		Multivariate analysis	
	HR (95% CI)	*p*-value	HR (95% CI)	*p*-value
Early ir-fatigue				
Yes vs no	2.25 (1.63–3.1)	< 0.0001	2.29 (1.62–3.22)	< 0.0001
Sex				
Male vs female	1.05 (0.78–1.41)	0.7515	–	–
Age at diagnosis				
Elderly vs non-elderly	1.20 (0.91–1.58)	0.1940	–	–
Primary tumor (melanoma)				
NSCLC	1.51 (1.09–2.08)	0.0124	1.08 (0.76–1.53)	0.6446
Renal cell carcinoma	1.55 (1.06–2.28)	0.0241	1.22 (0.81–1.85)	0.3445
Others	1.38 (0.43–4.40)	0.5790	0.95 (0.29–3.06)	0.9336
Treatment line				
Non-first vs first	1.75 (1.28–2.42)	0.0005	1.66 (1.17–2.35)	0.0041

**Table 3:** Univariate and multivariate analysis of OS

Variable (comparator)	Overall survival			
	Univariate analysis		Multivariate analysis	
	HR (95% CI)	*p*-value	HR (95% CI)	*p*-value
Early ir-fatigue				
Yes vs no	2.63 (1.83–3.77)	< 0.0001	2.32 (1.59–3.38)	< 0.0001
Sex				
Male vs female	1.41 (0.97–2.05)	0.0669	–	–
Age at diagnosis				
Elderly vs non-elderly	1.35 (0.96–1.89)	0.0758	–	–
Primary tumor (melanoma)				
NSCLC	2.24 (1.52–3.28)	< 0.0001	1.72 (1.14–2.60)	0.0095
Renal cell carcinoma	1.62 (0.99–2.63)	0.0518	1.40 (0.82–2.37)	0.2101
Others	0.74 (0.10–5.38)	0.7679	0.58 (0.07–4.29)	0.5992
Treatment line				
Non-first vs first	1.58 (1.08–2.31)	0.0170	1.33 (0.88–2.01)	0.1717

Considering the analysis of melanoma and NSCLC cohorts, early and delayed ir-fatigue were significantly related with an ECOG-PS ≥ 2 in melanoma patients, while only early ir-fatigue in NSCLC patients (data not shown). However, in both melanoma and NSCLC patients only the early ir-fatigue was significantly related to shorter PFS and OS, while not the delayed ir-fatigue (data not shown).

## Discussion

Cancer patients fatigue is a well know but complex symptom. It is the most reported symptom and it is related to the disease itself, to systemic inflammation, psychological condition, nutritional alterations, treatments side effects, genetic predisposition, and much more [10, 11].

Recently, Weber et al. [12] have reported that high baseline serum interleukin-6 (IL-6) levels are associated with a shortened survival in melanoma patients receiving nivolumab alone or ipilimumab alone, within the CheckMate 064 trial population [13]. Also high baseline serum C-reactive protein (CRP) levels, which is inducible by IL-6 [14], were reported to be related to a shortened survival, but only in patients receiving nivolumab alone [12]. High baseline serum CRP levels revealed to be related to a shortened survival also in melanoma patients receiving ipilimumab, nivolumab or the combination, within the CheckMate 067 trial population [12, 15], and in melanoma patients treated with nivolumab or dacarbazine within the CheckMate 066 trial population [12, 16]. Moreover, Weber and colleagues reported that increasing levels of IL-6 at week 12 were related to disease response in patients receiving nivolumab alone or ipilimumab alone [12].

IL-6 is one of the core cytokines involved in the cytokines release syndrome (CRS), which can occur with various clinical pictures, ranging from mild flu-like symptoms (including fatigue) to severe life-threatening manifestations of the overshooting inflammatory response [17].

Considering the occurrence of fatigue during immunotherapy as an immune-related event, it is interesting to speculate about its underlying mechanisms. Assuming that the immune activation induced by the administration of PD-1/PD-L1 checkpoint inhibitors may led to mild forms of CRS, the clinical occurrence of fatigue during immunotherapy might be induced by increasing levels of cytokines responsible of CRS, including IL-6, and therefore it could be related to worse clinical outcomes. Correspondingly, common irAEs (such as cutaneous irAEs, endocrine irAEs and gastro-intestinal irAEs), for which different underlying mechanisms have been proposed [18, 19], might be considered a surrogate predictor of clinical benefit with ICIs.

In our study population early ir-fatigue seems to be a prognostic parameter rather than predictive. Indeed, it revealed to be an independent predictor for shortened PFS and OS, while was not significantly related to ORR. On the other hand, delayed ir-fatigue was not related to any of the measured clinical outcomes. The early ir-fatigue might be considered as a treatment related effect, unlike the delayed ir-fatigue, which could be related to the progressive worsening of clinical condition. However, to properly weighing our results we must take into account the prevalent role of PS and disease burden (number of metastatic sites). Despite the 12-weeks landmark selection, which served to minimize negative selection biases, we found a significantly association between early ir-fatigue and poorer PS (p < 0.0001), as like early ir-fatigue and disease burden (p = 0.0003). Therefore, we must not fail in considering that our analysis is flawed by this association, and of course patients who experienced early ir-fatigue had a worse outcome mainly because of the poorer clinical condition.

This analysis has several limitations; as abovementioned, the selection bias, which does not allow us to make any conclusive consideration, due to the association between poorer clinical condition (PS and disease burden) and early ir-fatigue. Moreover, also the reporting of early ir-fatigue in clinical practice was flawed, because the fatigue might be related to many causes [10, 11]. On the other hand, our study suffers from a positive selection bias due to the 12-weeks landmark selection. In our opinion, for a proper estimation it would be crucial a perspective evaluation of serum IL-6 levels together with the fatigue assessment.

## Conclusion

Our study seems to reveal that early ir-fatigue is a negative prognostic parameter. However, to proper weigh its role we must to consider the predominant role of performance status, which was related to early ir-fatigue in the study population.

## Data Availability

The datasets used during the present study are available from the corresponding author upon reasonable request.

## Abbreviations

IL-6: interleukin-6
CRP: C-reactive protein
CRS: cytokines release syndrome
IrAE(s): immune-related adverse event(s)
PD-1/PD/L1: programmed death-1/programmed death-ligand 1
ORR: objective response rate
PFS: progression free survival
OS: overall survival
CTCAE: common terminology criteria for adverse events
ECOG-PS: Easter Cooperative Oncology Group-Performance Status
NSCLC: non small cell lung cancer
HR: hazard ratio
CI: confidence interval
